# Supplementary material for: A 14 × 14 μm2 footprint polarization-encoded quantum controlled-NOT gate based on hybrid waveguide
Source: Nat Commun. 2016 May 4;7:11490. doi: 10.1038/ncomms11490 (PMC4857478; doi:10.1038/ncomms11490)
Supplement: Supplementary Information — Supplementary Figures 1-4, Supplementary Notes 1-4 and Supplementary References [file ncomms11490-s1.pdf]

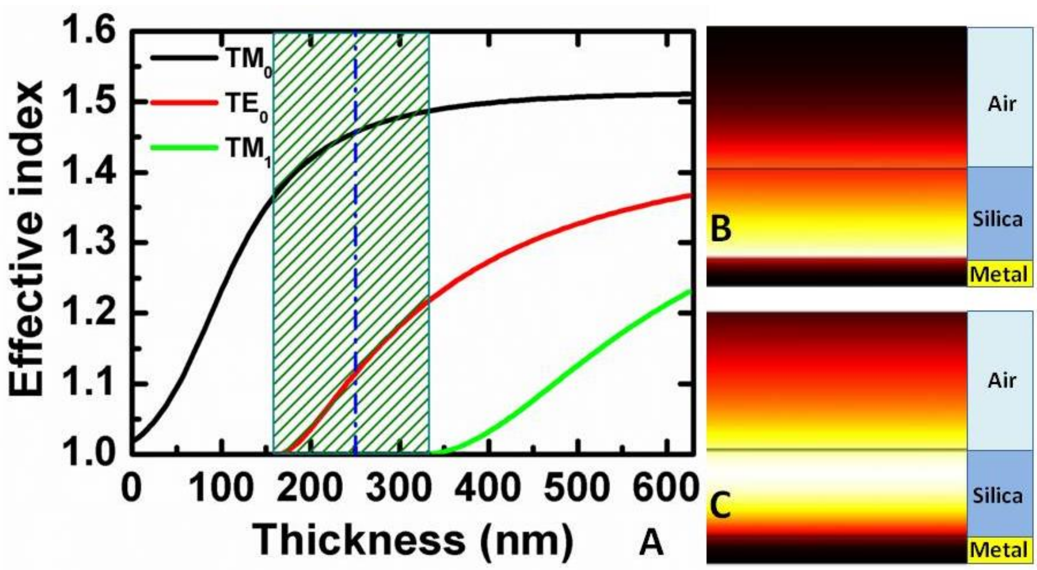

Supplementary Figure 1: Mode analysis of the HW waveguide system. (A) The dispersion relations of the waveguide modes in the HW system at 785 nm. (B) and (C) show the electric field distributions of the lowest TM (TM0, SPP) and TE (TE0) mode, respectively.

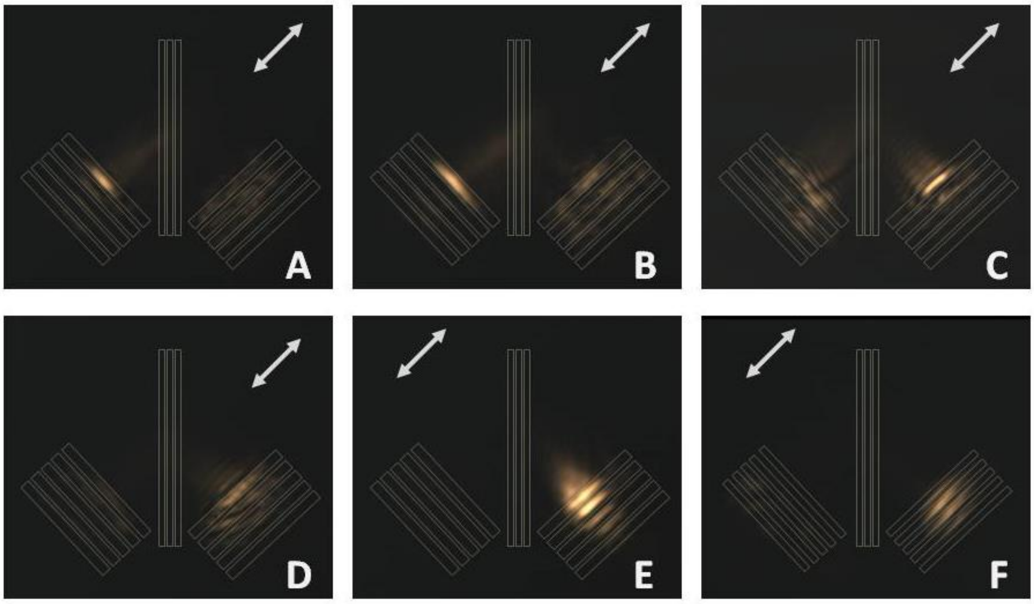

Supplementary Figure 2: **The classical characterization of PDBS with different parameters.** (A)-(D) show the SPP outputs with different milling depths of the reflector groove, with d=30 nm, 40 nm, 50 nm, and 60 nm, respectively. (E) and (F)

show the TE wave outputs with different period of the output slit of p=830 nm and 530 nm. The white arrows represent the areas of input lights and their polarizations.

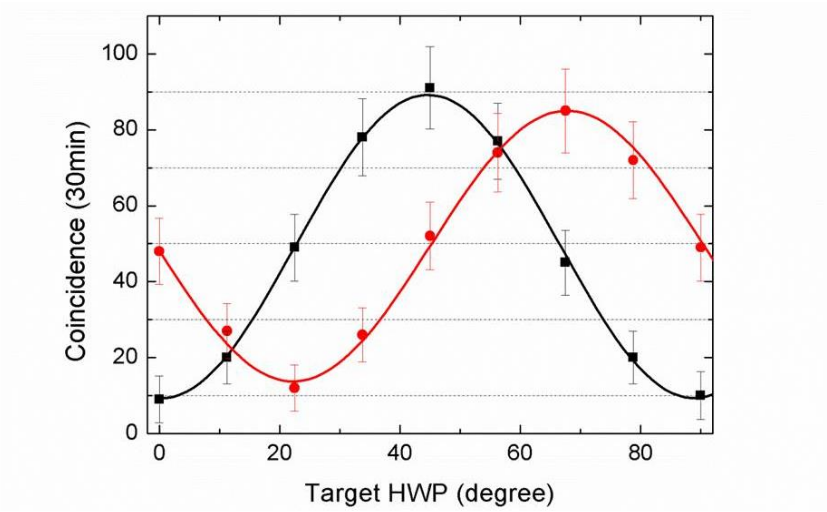

Supplementary Figure 3: Generation of polarization entanglement. Experimental results for producing a polarization entangled state from a separable input state. Polarization controller 2 was set to pass $|s\rangle$ (black dots) and $|s\rangle+|p\rangle$ (red dots), respectively, with lines fitting sine curves. Error bars are drawn to represent one standard deviation from the Poisson distribution.

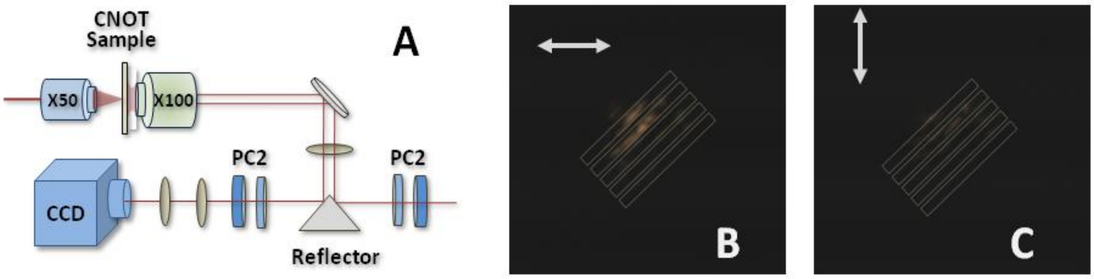

Supplementary Figure 4: Phase compensation and decoherence. (A) The schematic representation of the collection part of the setup, with one output field observed by a CCD camera. (B) and (C) show the CCD image of the output for (s +

p)-polarized (45º oblique to the output slits) polarization input and 45º /-45º collection, respectively.

Supplementary Note 1: Mode analysis of HW system

To guarantee only the lowest TE and TM (SPP) mode exist in the HW system, the dielectric-loaded SPP waveguide, the thickness of the loaded dielectric should be carefully chosen. We theoretically derive the dispersion relation of the TE and TM modes in the HW system against the thickness of the loaded dielectric **t**. The permittivity of silver is quoted from Ref. 1, and the index of silica is *1.5*. The dispersion relation of the system is shown in Supplementary Figure 1 A. The region in the green box in Supplementary Figure 1 A satisfies the requirement that only the lowest TE and TM modes simultaneously exist. We choose the thickness to be **t**=*250 nm* pointed by the blue dash dot line. The electric field distributions of the lowest TM (SPP) and TE mode are shown in Supplementary Figure 1 B and C. It is clear that their field locations are different and thus we can separately control these two modes in the HW-based gate.

Supplementary Note 2: TM reflector and TE transmitter

The SPP beam-splitter in the HW-based gate is the core part of the device, whose parameters should be carefully chosen. Among a large number of samples, we choose the three-groove grating with width *w=100 nm*, depth *d=50 nm*, and period *p=380 nm* to obtain the required *1:2* T/R ratio. The T/R ratio of the SPP can be easily tuned by

changing depth of the grooves. The outputs of the SPPs passing through the reflectors with different groove depths are presented in Supplementary Figure 2 A-D. For a deeper groove, the reflection of SPP will be larger. The width of the grooves has a less influence on the T/R ratio.

The output of TE mode can also be tuned by carefully choosing the parameters of the output slit. For required transmission of the TE mode, we choose the period of the output slit to be *830 nm*. Figure S2 E and F show the transmissions of the TE modes with different periods of output slits of **p**=*830 nm* and *530 nm*, respectively.

Supplementary Note 3: Polarization entanglement

The CNOT gate can be used as an entangling gate, which can produce a two-qubit entangled state from a separable input state. We input the CNOT gate the state $\left(|s\rangle_c - |p\rangle_c\right)|p\rangle_t \big/ \sqrt{2}$, and may produce a post-selected maximally entangled Bell state $|\psi^-\rangle = \left(|s,p\rangle - |p,s\rangle\right)\big/\sqrt{2}$ after the CNOT gate. By projecting the control output state to $|s\rangle_c$ and $\left(|s\rangle_c + |p\rangle_c\right)\big/\sqrt{2}$, respectively, we measured the coincidence as a function of the HWP setting in the target polarization controller and obtained two curves as shown in Supplementary Figure 3. The visibilities obtained by $V = (C_{max} - C_{min}) / (C_{max} + C_{min})$, are $84.4 \pm 9.0\%$ and $78.8 \pm 10.1\%$ for the two fitted curves, respectively, which are both above *78%* and hence such polarization-entangled state can violate Bell inequalities (2). Consequently, the high visibilities show a typical signature of entanglement creation of the gate.

Supplementary Note 4: Phase compensation and decoherence

Since the effective indexes of the TM (SPP) and TE mode in the HW system are different (*1.5* and *1.15*), the phase difference between these two polarized modes can be easily compensated by a phase compensator. The 45° input can be collected by a CCD camera with PC2 setting to 45° pass, as shown in Supplementary Figure 4 A. To see the purity of the output light, PC2 is set to 45° and -45° pass, corresponding to Supplementary Figure 4 (B) and (C), respectively. By using a well placed phase compensator, the output power ratio between 45° /-45° polarizations is about *10:1*, which is good enough for the CNOT gate (Supplementary Figure 4 B and C).

It should also be mentioned that the phase-damping decoherence of the photons generated from parametric down conversion could occur in such HW-based devices. However, we can see that the phase difference, about 4λ, is much smaller than the coherence length of the photon pair source, which is about 100λ, see Fig. 3 A. The coherence length can be further increased using a narrow-band photon-pair source, which meets the scalability requirement of the quantum chip. Therefore, the HW system is capable to be used in polarization encoding quantum information processing, as long as the phase difference is smaller than the coherence length.

Supplementary References:

1. P. B. Johnson and R. W. Christy, Optical Constants of the Noble Metals, *Phys. Rev. B* **6**, 4370-4379 (1972).

2. C. H. Bennett, *et al.*, Purification of Noisy Entanglement and Faithful Teleportation via Noisy Channels, *Phys. Rev. Lett.* **76**, 722-725 (1996).
